# Outcomes of endoscopic ultrasound-guided ablation and minimally invasive surgery in the treatment of pancreatic insulinoma: a systematic review and meta-analysis

**DOI:** 10.3389/fendo.2024.1367068

**Published:** 2024-04-05

**Authors:** Dan Xiao, Li Zhu, Si Xiong, Xu Yan, Qin Jiang, Ao Wang, Yegui Jia

**Affiliations:** ^1^ Department of Gastroenterology, The Sixth Hospital of Wuhan, Affiliated Hospital of Jianghan University, Wuhan, Hubei, China; ^2^ Gastrointestinal Surgery, The Sixth Hospital of Wuhan, Affiliated Hospital of Jianghan University, Wuhan, Hubei, China; ^3^ Department of Gastroenterology, Tongji Hospital, Tongji Medical College, Huazhong University of Science and Technology (HUST), Wuhan, Hubei, China; ^4^ Internal Medicine Department, University Hospital, Wuhan Institute of Technology, Wuhan, Hubei, China

**Keywords:** insulinoma, endoscopic ultrasound, ablation, minimally invasive surgery, adverse event, clinical outcomes

## Abstract

**Background and aims:**

Most pancreatic insulinomas can be treated by minimally invasive modalities. The aim of this meta-analysis was to assess the clinical outcomes of endoscopic ultrasound (EUS)-guided ablation and minimally invasive surgery (MIS) in the treatment of pancreatic insulinoma.

**Materials and methods:**

Online databases were searched for relevant studies. The primary aim was to compare the rates of adverse events (AEs) and the secondary aims were to compare the clinical and technical success rates, length of hospital stays, and symptom recurrence rates between EUS and MIS approaches.

**Results:**

Eight studies with 150 patients were identified that reported EUS-guided ablation outcomes, forming the EUS group, and 9 studies with 236 patients reported MIS outcomes, forming the MIS group. The pooled median age of the included patients in the EUS group was greater than that of the MIS group (64.06 vs. 44.98 years old, *p* < 0.001). Also, the technical success rate was significantly higher in the EUS group (100% vs. 96.6%, *p =* 0.025), while the clinical success was significantly higher (6%) in the MIS group (94% vs. 98.7%, *p =* 0.021). The AE rates (18.7% vs. 31.1%, *p =* 0.012) and severe AE rates (1.3% vs. 7.9%, *p =* 0.011) were significantly lower in the EUS group. The median length of hospital stay in the EUS group (2.68 days, 95% CI: 1.88–3.48, I^2 = ^60.3%) was significantly shorter than in the MIS group (7.40 days, 95% CI: 6.22–8.58, I^2 = ^42.2%, *p* < 0.001). The recurrence rate was significantly higher in the EUS group (15.3% vs. 1.3%, *p* < 0.001).

**Conclusions:**

EUS-guided ablation is associated with a lower AE rate and a shorter length of hospital stay, but a higher recurrence rate for the treatment of insulinoma compared with MIS. The EUS approach may be an alternative, even first-line, treatment for poor surgery candidates.

## 1 Introduction

Pancreatic neuroendocrine tumors (panNETs) account for less than 2% of all pancreatic tumors (1). According to the presence or absence of a clinical hormonal hypersecretion syndrome, panNETs are classified into functional or non-functional tumors. The most prevalent functional panNET is insulinoma (2). Insulin hypersecretion and hypoglycemia, which are associated with hypoglycemic, neuroglycopenic, and sympathetic-overstimulation symptoms, are the main manifestations of insulinoma (1). The early occurrence of obvious clinical symptoms of insulinoma generally allows its early diagnosis, when the insulinoma will still be small, commonly ranging in size from 5 to 20 mm at the time of early diagnosis (3).

Most insulinomas are benign single tumors, and surgical resection is the main treatment modality (4). However, there is a considerable risk of adverse events (AEs) with pancreatic surgery. A systematic review with 62 studies indicated that postoperative pancreatic fistula, delayed gastric emptying (DGE), and hemorrhage occurred in 14%–58%, 5%–18%, and 1%–7% of panNET cases after surgery, respectively, and even in-hospital death in 3%–6% of patients (5).

Therefore, alternative therapy modalities with a less invasive nature have attracted increasing attention and some have been used in clinical settings. Minimally invasive surgery (MIS), including laparoscopic and robotic surgery for insulinomas, has been reported to be associated with a lower incidence of AEs, shorter hospital stays, and a similar treatment efficacy when compared with open surgery (6). Recently, endoscopic ultrasound (EUS)-guided ablation, including radiofrequency ablation (RFA) and ethanol ablation (EA), has been reported. Considering the generally small size and benign nature of insulinomas, the endoscopic approach may be an optimal alternative to surgical resection. However, there are scant studies comparing the outcomes of EUS-RFA, EUS-EA, and surgery, especially minimally invasive surgery.

To fill this gap, we conducted a systematic review and meta-analysis. The primary aim was to compare the rates of adverse events (AEs) and the secondary aims were to compare the clinical and technical success rates, length of hospital stays, and symptom recurrence rates between EUS and MIS approaches.

## 2 Materials and methods

### 2.1 Search strategy

The Preferred Reporting Items for Systematic Reviews and Meta-Analyses (PRISMA) guidelines were followed for conducting this meta-analysis. Through systematic searches of the PubMed, Cochrane Library, and Web of Science databases, we were able to retrieve literature in English that had been published from the time the databases were created until December 1, 2023. We used the following Medical Subject Heading (MeSH) terms to search the literature in the aforementioned databases: “insulinoma,” “endoscopic ultrasound,” “radiofrequency ablation,” “ethanol ablation,” “minimally invasive surgery,” “laparoscopic surgery,” and “robotic surgery.” Only articles in English were searched and checked.

### 2.2 Selection criteria

The study inclusion criteria were as follows (1): clinical studies with human patients; (2) patients diagnosed with insulinoma treated with EUS-guided ablation or MIS; and (3) studies where the AEs, clinical and technical success rates, length of hospital stays, and symptom recurrence rates were reported; (4) studies that were classed as medium and high quality according to the Newcastle–Ottawa scale (NOS).

The exclusion criteria were as follows: (1) editorials, letters, reviews, meta-analyses, protocols, and case reports; (2) no detailed results were provided or the outcomes were not clear; (3) insulinomas were contaminated with other panNETs; and (4) duplicate studies. Finally, a full-text check was conducted to examine whether the identified papers met the inclusion criteria and passed the exclusion criteria. Two independent researchers performed the above processes, and their search results were consistent.

### 2.3 Quality assessment

We used the NOS as an assessment indicator since most of the relevant research in the studies was retrospective or single-arm. Studies with an NOS rating of 7–9 were considered high quality, while those with an NOS rating of 4–6 were considered medium quality.

### 2.4 Data extraction

Two independent researchers extracted the data from the included papers. If disagreements existed, they were resolved by the other co-authors. The following data were extracted: last name of the author, year of publication, study country, ages of the patients, number of patients with insulinoma, treatment methods, AEs, clinical and technical success rates, length of hospital stays, and symptom recurrence rates. A severe AE was defined as an AE that needed re-intervention, or had a Clavien-Dindo classification ≥ III.

Clinical success was defined as the recovery from insulinoma-associated symptoms. Symptom recurrence was defined as the recurrence of insulinoma-associated symptoms.

### 2.5 Statistical analysis

The primary aim was to compare the AEs between the EUS and MIS approaches. The secondary aims were to compare the clinical and technical success rates, length of hospital stays, and symptom recurrence rates between EUS and MIS approaches. The above endpoint proportions were pooled and analyzed. The I^2^ value was used to assess heterogeneity between the studies. A random effect result was used with an otherwise fixed-effect outcome, with I^2^ > 50% deemed significantly heterogeneous. Sensitivity analysis was used to find the potential study that could cause significant heterogeneity. Visual examination of the funnel plot and quantitative analysis utilizing Egger’s test of the intercept were used to evaluate publication biases. All the statistical analyses were conducted with Stata (Version 14). A *p-value* of 0.05 was considered statistically significant.

## 3 Results

### 3.1 Search results

After searching the aforementioned databases, 63 studies were identified that reported EUS-guided ablation for insulinoma, and 84 studies reported MIS for insulinoma.

**Figure 1:**
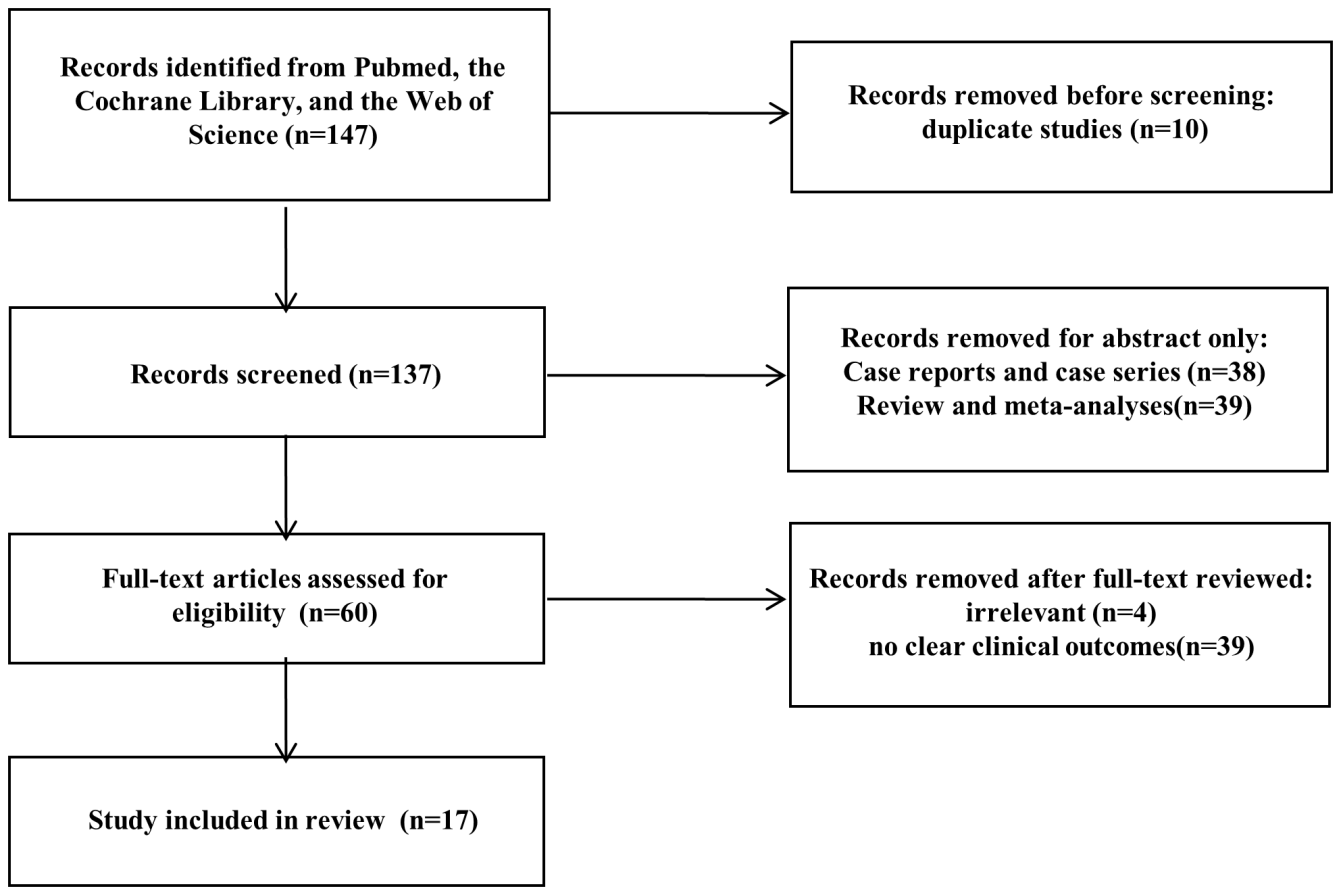
Preferred Reporting Items for Systematic Reviews and Meta-Analyses (PRISMA) flow diagram showing the study selection process.

Among the EUS studies, 5 were duplicate studies, 23 studies were case reports or case series reports with a sample size ≤ 5, 18 studies were reviews and meta-analyses, 2 studies were irrelevant, and 7 studies had no clinical outcomes. Finally, 8 studies with 150 patients were included in the data analysis (**Figure 1**) (7–14).

Among the MIS studies, 5 were duplicate studies, 15 studies were case reports or case series reports with a sample size ≤ 5, 21 studies were reviews and meta-analyses, 2 studies were irrelevant, and 32 studies had no clear clinical outcomes for laparoscopic surgery. Finally, 9 studies with 236 patients were included in the data analysis (15–23).

All the studies were conducted in referral centers, and the main indications for patients choosing EUS-RFA were that they were not good candidates for surgery or were unwilling to undergo surgery.

### 3.2 Quality assessment

All 17 studies identified in the initial screening mentioned above were retrospective studies, and they all underwent a quality appraisal using the NOS system (**Supplementary Table 1**) by two independent authors. Among these, 11 studies were assessed as medium quality, and six studies were assessed as high quality, according to the NOS scale, and thus passed the quality criterion. Consequently, the 17 studies were all included in our meta-analysis. The patient characteristics and study endpoints of the included studies are presented in **Tables 1**, **2**, respectively.

**Table 1:** Characteristics of the included studies.

Author	Publication year	Study design	Country	Total patients, n	Number of lesions, n	Treatment method	Median age (range), y	Median tumor size, mm (range)	Tumor location, (H/N/B/T)	Tumor grade, (G1/G2)
Debraine et al. (7)	2023	Retrospective	Belgium	11	11	RFA	65 (49–84)	11.6 (6.0-22.0)	5/1/3/2	9/2
Oleinikov et al. (8)	2019	Retrospective	Israel	7	9	RFA	60 (28-82)	13.0 (12.0-19.0)	7/0/2/0	9/0
Marx et al. (9)	2021	Retrospective	Switzerland	7	7	RFA	66 (48-97)	13.0 (8.0-20.0)	1/3/2/1	4/1
Sada et al. (10)	2023	Retrospective	US	8	8	EA	69.5 (34-84)	17.0 (12.1, 21.0)	4/1/1/2	NR
Yan et al. (11)	2022	Retrospective	China	9	10	EA	60 (32-69)	17.0 (11.0-21.0)	7/0/2/1	NR
Andreis et al. (12)	2023	Retrospective	Italy	10	10	RFA	65.5 (51-84)	11.0 (8.0-19.0)	3/0/3/4	9/1
Jürgensen et al. (13)	2023	Retrospective	Germany	9	9	EA	68 (57-79)	14.0 (7.0-21.0)	1/0/5/3	NR
Crinò et al. (14)	2023	Retrospective	Italy	89	89	RFA	55.0 (39-71)	13.0 (9.0-21.0)	34/0/39/16	66/3*
Espan˜a-Go´mez et al. (15)	2009	Retrospective	Mexico	14	14	Laparoscopic	42 (28-56)	20.0 (11.0–52.0)	1/1/7/5	NR
Belfiori et al. (16)	2018	Retrospective	Italy	15	15	MIC-EN	39 (24–77)	12.5 (9–26)	4/0/5/6	NR
Cunha et al. (17)	2007	Retrospective	France	12	12	Laparoscopic	48 (29-67)	13.0 (7.5-18.5)	2/4/1/5	NR
Hu et al. (18)	2011	Retrospective	China	43	43	Laparoscopic	42 (27.5-56.5)	14.5 (8.0-21)	9/10/12/12	NR
Isla et al. (19)	2007	Retrospective	UK	21	21	Laparoscopic	46 (22–70)	NR	5/0/9/7	NR
Nakamura et al. (20)	2015	Retrospective	Japan	15	16	Laparoscopic	57 (39-75)	16.0 (9.0-23.0)	3/0/5/8	14/2
Yin et al. (21)	2023	Retrospective	China	85	85	36 with laparoscopic and 49 with robotic	45 (32.5-57.5) and 49 (37-61)	17.0 (13.0-20.0) and 15.0 (13.0-20.0)	17/18/25/25	41/44
Roland et al. (22)	2008	Retrospective	USA	22	22	Laparoscopic	NR	NR	NR	NR
Sciuto et al. (23)	2014	Retrospective	Italy	9	10	Laparoscopic	36 (28-59)	18.0 (15.0-32.0)	0/0/4/6	NR

RFA, radiofrequency ablation; EA, ethanol ablation; NR, not reported; H/N/B/T, head/neck/body/tail; MIC-EN, mini-invasive enucleation.

*The tumor grade was unknown for the rest of the 20 patients.

**Table 2:** Study endpoints of the included studies.

Author	Publication year	Study design	Country	Total patients, n	Number of lesions, n	Treatment approach	Severe AEs, n (%)	Mild AEs, n (%)	Technical success rate, %	Clinical success rate, %	Length of hospital stay days	Median follow-up period, month (range)	Symptom recurrence rates, n%
Debraine et al. (7)	2023	Retrospective	Belgium	11	11	RFA	0	4 (36.4)	100	90.9	NR	26 (9-53)	0
Oleinikov et al. (8)	2019	Retrospective	Israel	7	9	RFA	0	0	100	100	NR	9 (3-21)	0
Marx et al. (9)	2021	Retrospective	Switzerland	7	7	RFA	1 (14)	3 (43)	100	100	2.0 (1.0-3.0)	21 (3-38)	0
Sada et al. (10)	2023	Retrospective	US	8	8	EA	0	0	100	75	NR	43 (19.5, 81.5)	2 (25)
Yan et al. (11)	2022	Retrospective	China	9	10	EA	1 (7.1)	0	100	77.8	NR	33 (1-52)	5 (55.6)
Andreis et al. (12)	2023	Retrospective	Italy	10	10	RFA	0	2 (20)	100	100	NR	19.5 (12-59)	0
Jürgensen et al. (13)	2023	Retrospective	Germany	9	9	EA	0	1 (11.1)	100	100	4.0 (2.0-5.0)	17 (1-35)	1 (11.1)
Crinò et al. (14)	2023	Retrospective	Italy	89	89	RFA	0	16 (18.0)	100	95.5	3.5 (0.5-6.5)	23 (14-31)	15 (16.8)
Espan˜a-Go´mez et al. (15)	2009	Retrospective	Mexico	14	14	Laparoscopic	0	9 (64.3)	100	100	10.0 (2-21)	42 (1-90)	0
Belfiori et al. (16)	2018	Retrospective	Italy	15	15	MIC-EN	2 (13.3)	8 (53.4)	100	100	9 (5-54)	41 (1–134)	1 (6.7)
Cunha et al. (17)	2007	Retrospective	France	12	12	Laparoscopic	1 (8.3)	3 (25)	100	91.7	13 (7-20)	49 (20-78)	0
Hu et al. (18)	2011	Retrospective	China	43	43	Laparoscopic	5 (11.6)	8 (18.6)	100	95.3	9.0 (3.5-14.5)	6	0
Isla et al. (19)	2007	Retrospective	UK	21	21	Laparoscopic	1 (4.8)	2 (9.5)	100	100	5 (1–18)	NR	0
Nakamura et al. (20)	2015	Retrospective	Japan	15	16	Laparoscopic	2 (13.3)	0	100	100	12 (7–63)	43 (3–88)	0
Yin et al. (21)	2023	Retrospective	China	85	85	36 with laparoscopic and 49 with robotic	4 (11.1) and 3 (6.2)	32 (90.6) and 45 (91.8)	94.1	100	8.5 (6.0-11.3) and 6.0 (4.0-7.0)	65 (1-159)	2 (2.4)
Roland et al. (22)	2008	Retrospective	USA	22	22	Laparoscopic	0	3 (13.6)	90.9	100	13.0 (8.5-17.5)	38.0 (33-43)	0
Sciuto et al. (23)	2014	Retrospective	Italy	9	10	Laparoscopic	1 (11.1)	2 (22.2)	100	100	7.0 (5-18)	45 (11-72)	0

RFA, radiofrequency ablation; EA, ethanol ablation; NR, not reported; AEs, adverse events.

### 3.3 Patient characteristics

In total, 391 lesions were identified from the 386 patients included in the 17 studies in the meta-analysis. Overall, 150 patients underwent EUS-guided ablation, whereby 26 (17.3%) underwent EUS-EA and 124 (82.7%) underwent EUS-RFA. The other 236 patients underwent minimally invasive surgery, among whom 52 (22.0%) underwent robotic surgery and 184 (78.0%) underwent laparoscopic surgery.

The pooled median age of the included patients was 53.05 years old (range: 22–97 years old); patients in the EUS group were older than those in the MIS group (64.06 vs. 44.98 years old, *p* < 0.001). Except for two studies (19, 22), the tumor size was reported in 15 studies. The pooled overall median tumor size was 14.74 mm (ranging from 3–52 mm), with no significant difference identified between the EUS and MIS groups (14.05 vs. 15.46 mm, *p* = 0.303). Except for one study (22), the other 16 studies (representing 369 lesions) reported the tumor location. In the EUS group, 67 lesions (43.8%) were located in the pancreatic head and neck, 57 (37.3%) in the pancreatic body, and 29 (18.9%) in the pancreatic tail. In the MIS group, 74 lesions (34.3%) were located in the pancreatic head and neck, 68 (31.4%) in the pancreatic body, and 74 (34.3%) in the pancreatic tail. The tumor distribution showed no significant difference between the two groups (*p* = 0.063). However, only 5 studies (7–9, 12, 14) in the EUS group (comprising 97 grade 1 lesions and 5 grade 2 lesions) and 2 studies (20, 21) in the MIS group (comprising 55 grade 1 lesions and 46 grade 2 lesions) reported the tumor grade; however, among those, the proportion of grade 1 lesions was significantly higher in the EUS group (*p* < 0.001) (**Table 3**).

**Table 3:** Baseline characteristics and treatment outcomes of the included studies.

	EUS group	MIS group	*P-value*
Age, median years (range)	64.06 (28-97)	44.98 (22-84)	<0.001
Tumor size, median mm (range)	14.05 (6-21)	15.46 (7.5-52)	0.303
Tumor location, HN/BT, n	67/86	74/142	0.063
Tumor grade, Grade 1/2, n	97/5	55/46	<0.001
Adverse events, n (%)	28 (18.7)	47 (31.1)	0.012
Severe adverse events, n (%)	2 (1.3)	12 (7.9)	0.011
Technical success, n (%)	150 (100)	228 (96.6)	0.025
Clinical success, n (%)	141 (94)	3 (98.7)	0.021

H/N/B/T, head/neck/body/tail; MIS, minimally invasive surgery.

### 3.4 Treatment-related adverse events

There was an unexpectedly high AE rate (98.8%) reported in the study by Yin et al. (21), and this was determined to be the origin of the heterogeneity in this analysis, so the study was removed from the further analysis of the AEs described below.

In the EUS group, 28 AEs (18.7%) occurred in 150 patients, including 2 severe AEs (1.3%). In the MIS group, 47 AEs (31.1%) occurred in 151 patients, including 12 severe AEs (7.9%). The AE rates and severe AE rates were significantly lower in the EUS group compared with the MIS group (*p* = 0.012 and 0.011) (**Table 3**).

### 3.5 Treatment outcomes

The technical success rate in the EUS group was 100%. However, 8 patients (3.4%) in the MIS group transferred to open surgery due to tumor location failure. The technical success rate in the MIS group was 96.6%. The technical success rate was significantly higher in the EUS group (*p =* 0.025). In terms of clinical success, 9 patients (6%) in the EUS group showed no symptom improvement after treatment, while 3 patients (1.3%) in the surgery group showed no symptom improvement after treatment. The difference between the two groups showed a statistical difference (*p* = 0.021) (**Table 3**).

**Figure 2:**
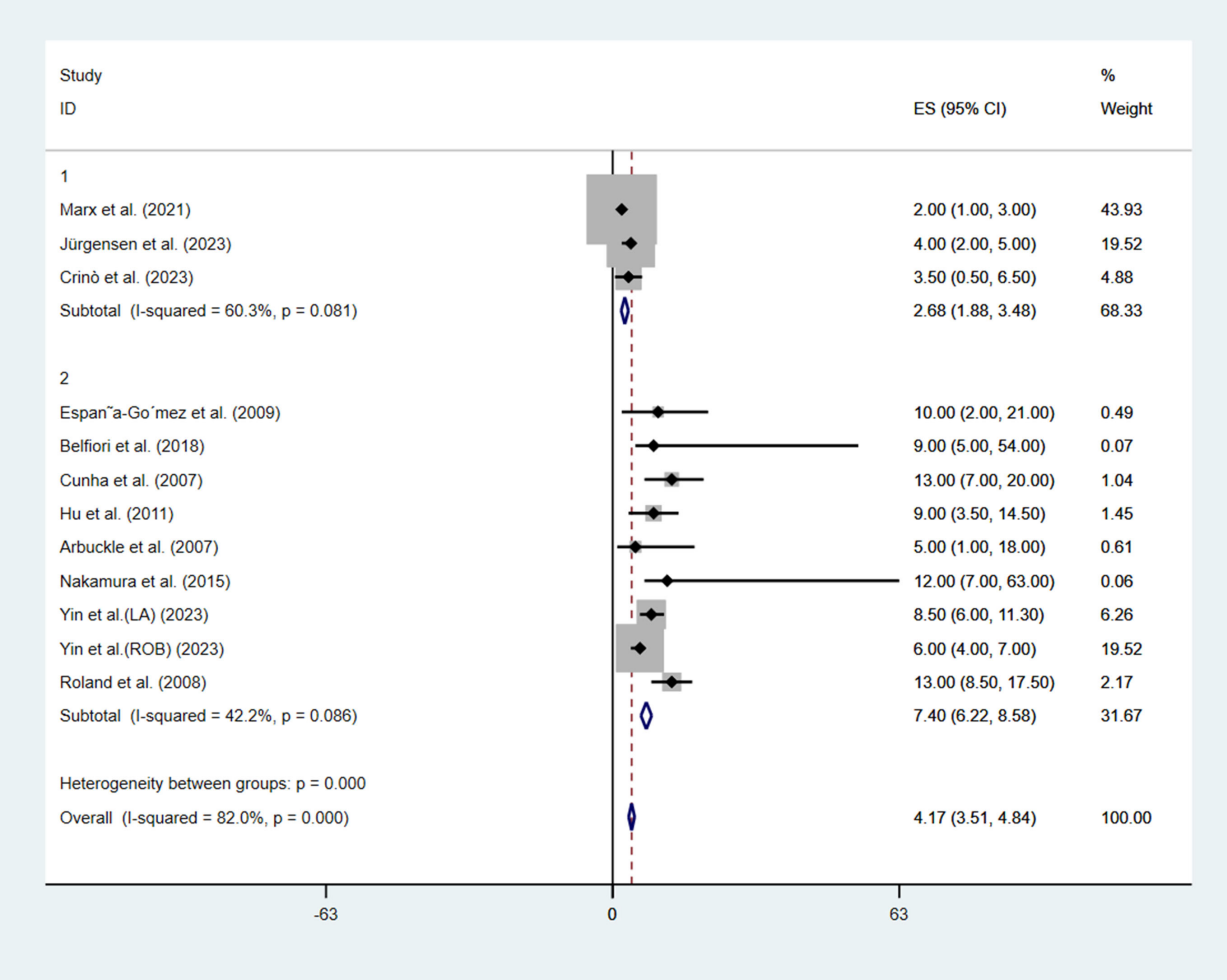
Pooled analysis of the length of hospital stay grouped by treatment method.

Regarding hospital stay after treatment, only 3 studies (9, 13, 14) in the EUS group reported the length of hospital stay, while all the studies in the MIS group reported the length of hospital stay. The median length of hospital stay in the EUS group (2.68 days, 95%CI: 1.88–3.48, I^2 = ^60.3%) was significantly shorter than that in the MIS group (7.40 days, 95%CI: 6.22–8.58, I^2 = ^42.2%), with a *p-value* < 0.001 (**Figure 2**).

The pooled median follow-up time in the EUS group was 18.91 months (ranging from 1–81.5 months), while the pooled median follow-up time in the MIS group was 24.62 months (ranging from 1–159 months). The median follow-up time was similar in the two groups with no significant difference (*p* = 0.068) (**Supplementary Figure 1**). Also, 23 cases (15.3%) in the EUS group experienced symptom recurrence, but only 3 cases (1.3%) experienced recurrence in the MIS group. The recurrence rate was thus significantly lower in the MIS group (*p* < 0.001).

### 3.6 Sensitivity analysis

Sensitivity analysis was next performed to evaluate the stability of the AE results. According to the results of the sensitivity analysis (**Supplementary Figures 2A, B**), except for the study by Yin et al. (21), no study needed to be removed to maintain the stability of the results for the AEs rates in the EUS group (**Supplementary Figure 2A**) or the MIS group (**Supplementary Figure 2B**). We thus believe the results for the AE rates were stable.

### 3.7 Publication bias

**Figure 3:**
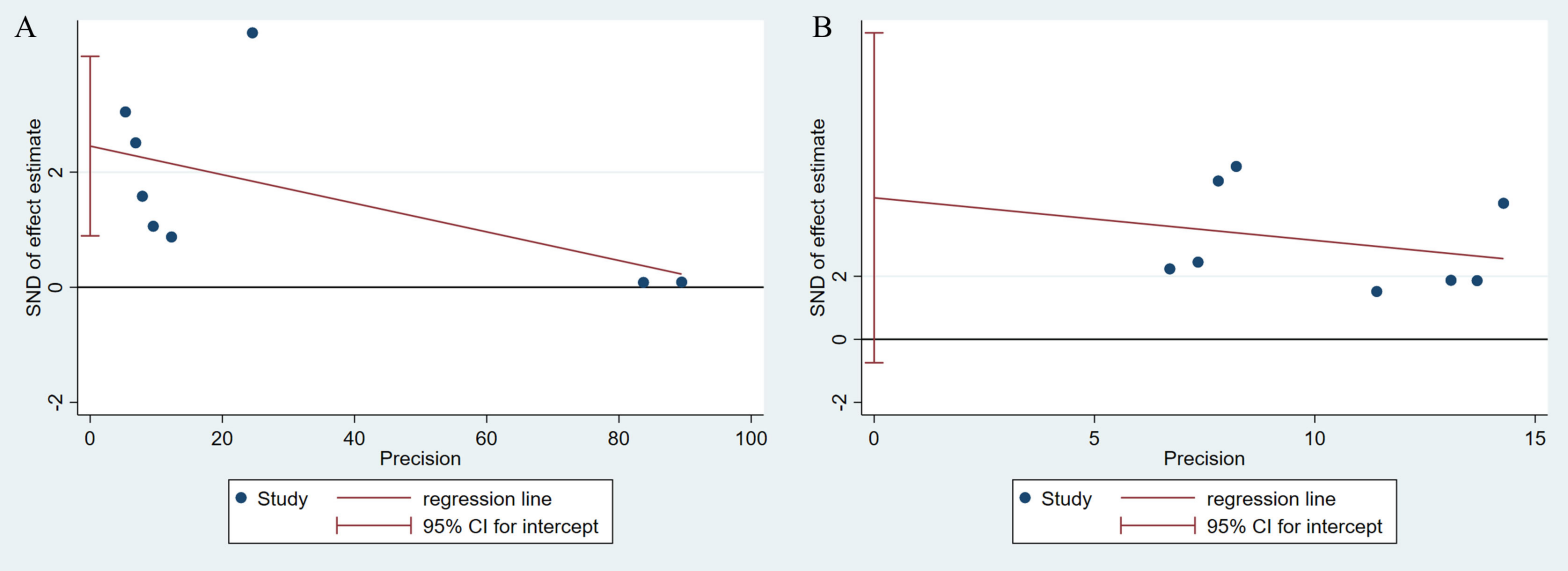
Egger’s tests were conducted for assessing the rates of adverse events to identify potential publication bias. **(A)** EUS group, **(B)** Minimally invasive group (*p = 0.082*, **(B)**.

Egger’s tests were conducted for the reported AE rates to identify any potential publication bias. A possibility of publication bias (*p* = 0.008, **Figure 3A**) was indeed identified as related to the AE rates in the EUS group. However, the AE rate showed no publication bias in the MIS group (*p* = 0.082, **Figure 3B**).

## 4 Discussion

In this meta-analysis, the treatment outcomes of EUS-guided ablation and MIS were presented and compared. We confirmed that EUS-guided ablation was associated with fewer incidences of AEs, a shorter length of hospital stay, and a higher technical success rate. Although recurrence rate was significantly higher in the EUS group, the patients were much older in that group and therefore poorer surgical candidates, and so we consider EUS-guided ablation to be a suitable alternative treatment approach for these and other poor surgical candidates.

Among all the panNETs, insulinomas are likely the best candidates for EUS-guided ablation because of their tiny size, minimal propensity for malignancy, and extremely quick symptom alleviation, which together make it simpler to track the effectiveness of treatment. However, EUS-guided ablation should not be performed in all insulinoma cases. Sporadic solitary insulinomas with a diameter of less than or equal to 20 mm, a minimum distance of 1 mm from the main pancreatic duct, and a Ki-67 value of less than 5% on EUS-guided cytology or from a biopsy sample may be the best candidates for this procedure, which was the condition considered in this meta-analysis. Moreover, the tumor sizes and tumor grades were found to be comparable between the EUS and MIS groups in this meta-analysis. The similar baseline characteristics of the included lesions indicate the results of this study are reliable.

The MIS treatment of insulinoma has gained widespread acceptance in the past decades. According to a meta-analysis, treating insulinomas through the laparoscopic approach is linked to a shorter hospital stay and comparable rates of postoperative AEs compared to open surgery (24, 25). However, for tumors located in the pancreatic head, pancreaticoduodenectomy is difficult to perform under MIS (26). Furthermore, to ensure full excision of the tumor, it is crucial to accurately identify the pancreatic resection line. As a result, guidance techniques, such as intraoperative ultrasound, should be used during MIS procedures (27). Considering this, additional expenses and operating time are required to provide proper surgical guidance. However, these limitations can be overcome by the use of EUS-guided ablation, whereby the tumor can be treated under real-time EUS guidance. Another meta-analysis concluded that the pooled sensitivity, specificity, and area under the ROC were 81%, 90%, and 0.92 for this approach (28). The results of that study indicated that EUS was an accurate approach for the preoperative localization of insulinomas (28). In our study, no difference in tumor distribution was observed. However, the *p-value* was very close to 0.05 (0.063), and the reported tumor locations may have been influenced by the subjective judgments of the radiologists and surgeons. We can conclude that for insulinomas located in the pancreatic head or neck, EUS-guided ablation is usually the preferred treatment method.

In 1999, Goldberg et al. (29) reported the first experimental EUS-RFA procedure in a pig model. They forecast the possible future application of EUS-RFA: “The development of endosonographically placed therapeutic devices may provide a unique alternative for the management of premalignant pancreatic lesions and potentially may offer palliative therapy for surgically unresectable malignant pancreatic tumors.” In 2006, Jürgensen et al. first reported the use of EUS-EA in treating an insulinoma, in which a 78-year-old patient achieved a durable, complete remission of their tumor (30). Several case reports and case series reports have confirmed the potential advantages of EUS-guided ablation in treating insulinomas. Recently, a meta-analysis with 19 studies and 183 patients (comprising 101 functional panNETs and 95 non-functional panNETs) summarized that the pooled overall AE rates for clinical efficacy were 17.8% and 95.1% for functional panNETs and 24.6% and 93.4% for non-functional panNETs. These results were very similar to those in our study. Another meta-analysis explored the safety and efficacy of EUS-guided ablation for solid pancreatic tumors. The AE rates were 32.2% for RFA and 21.2% (95% CI: 6.8–49.9%) for EA (31), and severe complications rarely occurred. However, the studies included in those meta-analyses were all single-arm studies. Comparative studies are required to verify the true value of EUS-guided ablation and MIS in the treatment of insulinomas. To the best of our knowledge, our study is the first comparative meta-analysis to compare the clinical outcomes of EUS and MIS approaches for treating insulinomas.

The high recurrence rate associated with EUS-guided ablation may be a concern for its wider clinical application. The reason for this high recurrence rate may be due to the fact that endoscopic ablation is more likely to have residual tumor cells than surgical resection, but no study has yet confirmed this hypothesis. Regarding the recurrence or advancement of insulinomas treated with EUS-guided ablation, the long-term results are still unclear. Our study first reported the long-term outcomes of EUS-guided ablation for the treatment of insulinoma. The recurrence rate was 15.6% after a median 18-month follow-up. Considering the recurrence rate was acceptable and the procedure can be repeated after recurrence, although the recurrence rate was higher, we still believe that EUS-guided ablation is a valuable approach. However, significant heterogeneity was identified among the different studies in our meta-analysis. Prospective studies with a larger sample size are warranted to verify the true long-term outcomes of EUS-guided ablation.

Undoubtedly, there were still some limitations of our study to note. First, the heterogeneity between studies caused by the methodological and clinical diversities was high. All the included studies were retrospective, so significant selection bias may exist. Second, all the studies compared the two treatment methods directly. The superiority of the two treatment methods should be verified in a further prospective randomized controlled study. Third, the different outcomes of EUS-RFA and EUS-EA were not analyzed due to the small sample sizes. However, the treatment mechanisms are different between EUS-RFA and EUS-EA, and the outcomes may be different. Fourth, the expression of data among different studies was different. Some studies expressed data as the median, and some expressed data as the mean. We estimated some baseline data to make the expressions consistent, which may have caused bias. Fifth, publication bias still existed in our study. The overwhelming predilection of sponsors, periodicals, and researchers to look for optimal outcomes was the main source of this publication bias. Furthermore, another element that contributed to publication bias was the considerable between-study heterogeneity. Sixth, although not statistically significant, the follow-up time was shorter in the EUS group in our study, which could have influenced the relapse rate.

## 5 Conclusion

We conducted a meta-analysis and systematic review to compare the treatment outcomes of EUS-guided ablation and MIS for the treatment of insulinoma. We identified that the age of the patients in EUS-guided ablation was associated with lower AE rates and lower severe AE rates, and a shorter length of hospital stay, but a higher recurrence rate after treatment. EUS-guided ablation may be an alternative, and even first-line, treatment for poor surgery candidates. Further prospective studies comparing the two treatment methods are warranted to establish the true role of EUS-guided ablation in the treatment of insulinomas.

## Data availability statement

The raw data supporting the conclusions of this article will be made available by the authors, without undue reservation.

## Author contributions

DX: Conceptualization, Formal analysis, Writing – original draft. LZ: Data curation, Formal analysis, Software, Writing – review & editing. SX: Formal analysis, Validation, Writing – original draft. XY: Data curation, Resources, Writing – review & editing. QJ: Formal analysis, Writing – original draft. AW: Writing – review & editing. YJ: Conceptualization, Writing – review & editing.

## Supplementary material

Supplementary Figure 1 Pooled analysis of the median follow-up time grouped by treatment method.

Supplementary Figure 2 Sensitivity analysis results of adverse events. **(A)** EUS-guided ablation, **(B)** Minimally invasive surgery.
