# Supplementary figures and images for: Outcomes of endoscopic ultrasound-guided ablation and minimally invasive surgery in the treatment of pancreatic insulinoma: a systematic review and meta-analysis
Source: Front Endocrinol (Lausanne). 2024 Apr 5;15:1367068. doi: 10.3389/fendo.2024.1367068 (PMC11026617; doi:10.3389/fendo.2024.1367068)

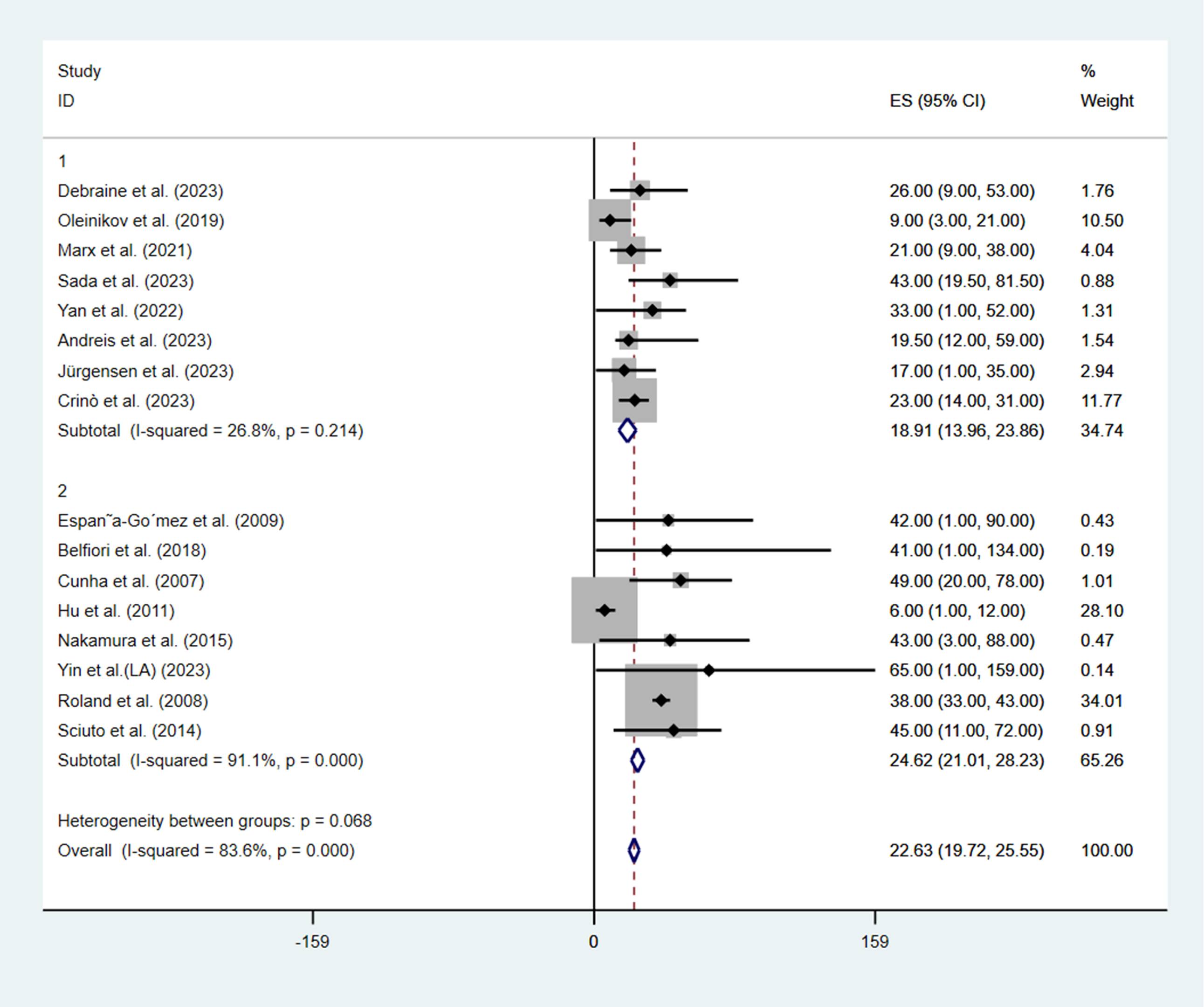

Supplement: Supplementary Figure 1 — Pooled analysis of the median follow-up time grouped by treatment method. [file Image_1.tif]

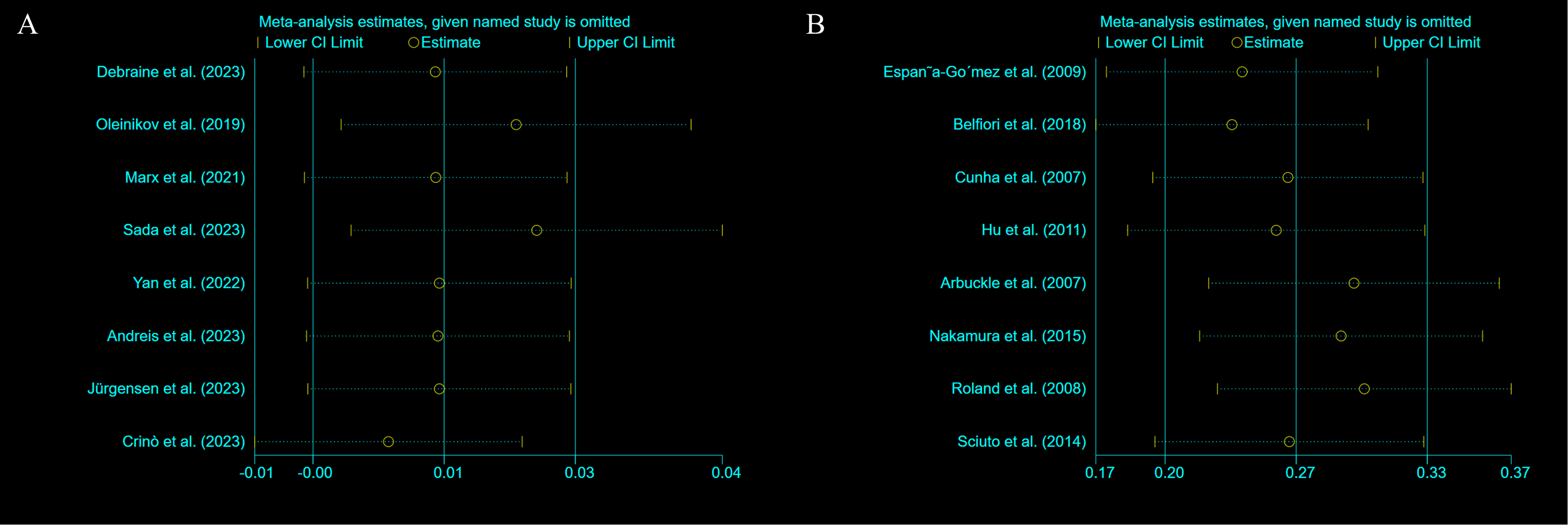

Supplement: Supplementary Figure 2 — Sensitivity analysis results of adverse events. (A) EUS-guided ablation, (B) Minimally invasive surgery. [file Image_2.tif]
