# Supplementary material for: Outcomes of endoscopic ultrasound-guided ablation and minimally invasive surgery in the treatment of pancreatic insulinoma: a systematic review and meta-analysis
Source: Front Endocrinol (Lausanne). 2024 Apr 5;15:1367068. doi: 10.3389/fendo.2024.1367068 (PMC11026617; doi:10.3389/fendo.2024.1367068)
Supplement: Supplementary file 3 [file Table_1.docx]

**Supplementary Table 1** The quality evaluations of all included articles according to Newcastle Ottawa Scale.

| **Author** | **Year** |  | **Selection** | | | |  | **Comparability** | |  | **Outcome** | | |  | **Total score** |
| --- | --- | --- | --- | --- | --- | --- | --- | --- | --- | --- | --- | --- | --- | --- | --- |
|  |  |  | **(1)** | **(2)** | **(3)** | **(4)** |  | **(1)** | **(2)** |  | **(1)** | **(2)** | **(3)** |  |  |
| Debraine et al. (7) | 2023 |  | **🟑** | - | **🟑** | **🟑** |  | - | - |  | **🟑** | **🟑** | **🟑** |  | 6 |
| Oleinikov  et al. (8) | 2019 |  | **🟑** | - | **🟑** | **🟑** |  | - | - |  | **🟑** | **🟑** | **🟑** |  | 6 |
| Marx et al. (9) | 2021 |  | **🟑** | - | **🟑** | **🟑** |  | - | - |  | **🟑** | **🟑** | **🟑** |  | 6 |
| Sada et al. (10) | 2023 |  | **🟑** | - | **🟑** | **🟑** |  | - | - |  | **🟑** | **🟑** | **🟑** |  | 6 |
| Yan et al. (11) | 2022 |  | **🟑** | - | **🟑** | **🟑** |  | - | - |  | **🟑** | **🟑** | **🟑** |  | 6 |
| Andreis et al. (12) | 2023 |  | **🟑** | - | **🟑** | **🟑** |  | - | - |  | **🟑** | **🟑** | **🟑** |  | 6 |
| Jürgensen et al. (13) | 2023 |  | **🟑** | **🟑** | **🟑** | **🟑** |  | **🟑** | - |  | **🟑** | **🟑** | **🟑** |  | 8 |
| Crinò et al. (14) | 2023 |  | **🟑** | **🟑** | **🟑** | **🟑** |  | **🟑** | - |  | **🟑** | **🟑** | **🟑** |  | 8 |
| Espan˜a-Go´mez et al. (15) | 2009 |  | **🟑** | - | **🟑** | **🟑** |  | - | - |  | **🟑** | **🟑** | **🟑** |  | 6 |
| Belfiori et al. (16) | 2018 |  | **🟑** | **🟑** | **🟑** | **🟑** |  | **🟑** | - |  | **🟑** | **🟑** | **🟑** |  | 8 |
| Cunha et al. (17) | 2007 |  | **🟑** | **🟑** | **🟑** | **🟑** |  | **🟑** | - |  | **🟑** | **🟑** | **🟑** |  | 8 |
| Hu et al. (18) | 2011 |  | **🟑** | **🟑** | **🟑** | **🟑** |  | **🟑** | - |  | **🟑** | **🟑** | **🟑** |  | 8 |
| Isla et al. (19) | 2007 |  | **🟑** | - | **🟑** | **🟑** |  | - | - |  | **🟑** | **🟑** | **🟑** |  | 6 |
| Nakamura et al. (20) | 2015 |  | **🟑** | - | **🟑** | **🟑** |  | - | - |  | **🟑** | **🟑** | **🟑** |  | 6 |
| Yin et al. (21) | 2023 |  | **🟑** | **🟑** | **🟑** | **🟑** |  | **🟑** | - |  | **🟑** | **🟑** | **🟑** |  | 8 |
| Roland et al. (22) | 2008 |  | **🟑** | **🟑** | **🟑** | **🟑** |  | **🟑** | - |  | - | **🟑** | **🟑** |  | 7 |
| Sciuto et al. (23) | 2014 |  | **🟑** | - | **🟑** | **🟑** |  | - | - |  | **🟑** | **🟑** | **🟑** |  | 6 |

**Selection 0-4🟑**

(1) Representativeness of the exposed cohort

(2) Selection of the non exposed cohort

(3) Ascertainment of exposure

(4) Demonstration that outcome of interest was not present at start of study

**Comparability 0-2🟑**

Comparability of cohorts on the basis of the design or analysis

(1) Study controls for _____________ (select the most important factor)

(2) Study controls for any additional factor (This criteria could be modified to indicate specific control for a second important factor.)

**Outcome 0-3🟑**

(1) Assessment of outcome

(2) Was follow-up long enough for outcomes to occur

(3) Adequacy of follow up of cohorts
